# Ni-mediated reactions in nanocrystalline diamond on Si substrates: the role of the oxide barrier

**DOI:** 10.1039/d0ra00809e

**Published:** 2020-02-26

**Authors:** Semir Tulić, Thomas Waitz, Oleksandr Romanyuk, Marián Varga, Mária Čaplovičová, Gerlinde Habler, Viliam Vretenár, Mário Kotlár, Alexander Kromka, Bohuslav Rezek, Viera Skákalová

**Affiliations:** Physics of Nanostructured Materials, Faculty of Physics, University of Vienna Boltzmanngasse 5 1090 Vienna Austria viera.skakalova@univie.ac.at; Institute of Physics, Czech Academy of Sciences Cukrovarnická 10 Prague 6 Czech Republic; Slovak University of Technology, Centre for Nanodiagnostics Vazovova 5 812 43 Bratislava Slovakia; Department of Lithospheric Research, University of Vienna Althanstrasse 14 1090 Vienna Austria; Faculty of Electrical Engineering, Czech Technical University Technická 2 Prague 6 Czech Republic

## Abstract

Nanocrystalline diamond (NCD) films grown on Si substrates by microwave plasma enhanced chemical vapor deposition (MWPECVD) were subjected to Ni-mediated graphitization to cover them with a conductive layer. Results of transmission electron microscopy including electron energy-loss spectroscopy of cross-sectional samples demonstrate that the oxide layer on Si substrates (∼5 nm native SiO_2_) has been damaged by microwave plasma during the early stage of NCD growth. During the heat treatment for graphitizing the NCD layer, the permeability or absence of the oxide barrier allow Ni nanoparticles to diffuse into the Si substrate and cause additional solid-state reactions producing pyramidal crystals of NiSi_2_ and SiC nanocrystals. The latter are found impinged into the NiSi_2_ pyramids but only when the interfacial oxide layer is absent, replaced by amorphous SiC. The complex phase morphology of the samples is also reflected in the temperature dependence of electrical conductivity, where multiple pathways of the electronic transport dominate in different temperature regions. We present models explaining the observed cascade of solid-state reactions and resulting electronic transport properties of such heterostructures.

## Abstract 2

Nanocrystalline diamond films grown on Si/native oxide substrates were subjected to Ni-mediated graphitization. Transmission electron microscopy study revealed crystals of NiSi_2_ and SiC across the carbon/silicon interface in addition.

## Graphical abstract

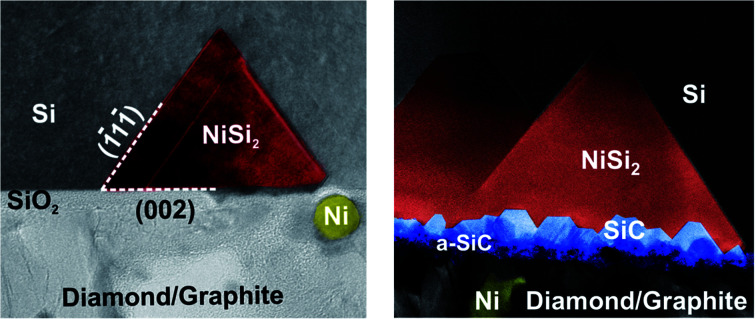

## Introduction

The growth of amorphous SiO_2_ layers on Si substrates led to their major breakthrough in the production of metal-oxide-semiconductor field-effect transistors and thus to a technological revolution.^1^ The oxide layers of several Angstroms to tens of nanometres mostly serve as the gate dielectric in transistors. In microelectronics fabrication, plasma processing plays an important role. Using plasma enhanced chemical vapor deposition (PECVD), Si also serves as a substrate for thin films of various compositions and thicknesses including nanocrystalline diamond (NCD). Plasma processing and so PECVD has a major drawback as plasma-induced surface damage can occur *e.g.* deteriorating the dielectric functionality of SiO_2_ layers.^2^ In order to prevent deterioration, the thickness of the oxide layer must be carefully designed. When NCD is grown on a Si substrate as a top dielectric layer, nevertheless, the damage to the oxide layer is unimportant since the electric insulating properties of NCD are superior to SiO_2_. Thus, Si wafers covered with native SiO_2_ (often ∼5 nm thick) are routinely used as substrates for NCD growth.^3^ In recent demonstrations of high-mobility graphene-based radio frequency field-effect transistors, instead of SiO_2_, graphene was transferred on such an NCD dielectric layer to avoid charge traps, always present in SiO_2_ hampering high performance of the device.^4^ However, when graphene is directly synthesized by Ni-mediated graphitization of NCD,^5–9^ actually, the graphitic layers are formed also in the bulk between the NCD nanocrystals.^10^ In such case, the quality of SiO_2_ interface, as we show here, can play an important role at the electronic transport. In this work, we present transmission electron microscopy (TEM) studies including high-resolution electron microscopy (HRTEM) and electron energy-loss spectroscopy (EELS) maps of cross-sectional NCD samples grown on Si substrates with a 5 nm thick native oxide layer. We then show PECVD conditions under which the oxide layer is preserved. More importantly, we also show that, in the absence of an oxide barrier at the interface between diamond and Si substrate, Si and C interdiffuse, resulting in formation of an amorphous SiC (a-SiC) layer. Introducing Ni atop of the NCD for the catalytic synthesis of graphene causes a cascade of solid-state reactions as Ni nanoparticles propagate towards the Si substrate: (1) graphitization of NCD, (2) formation of NiSi_2_ in the substrate, and (3) formation of crystalline SiC. We propose a model that describes these processes step-by-step during the thermal treatment of Ni/NCD/Si system. Finally, we measured the temperature dependence of the electrical conductivity; a rather complicated behaviour corresponds to multiple pathways of the electronic transport dominating different temperature regions.

## Results and discussion

### Structural and chemical analysis of NCD-1p and NCD-1

**Fig. 1:**
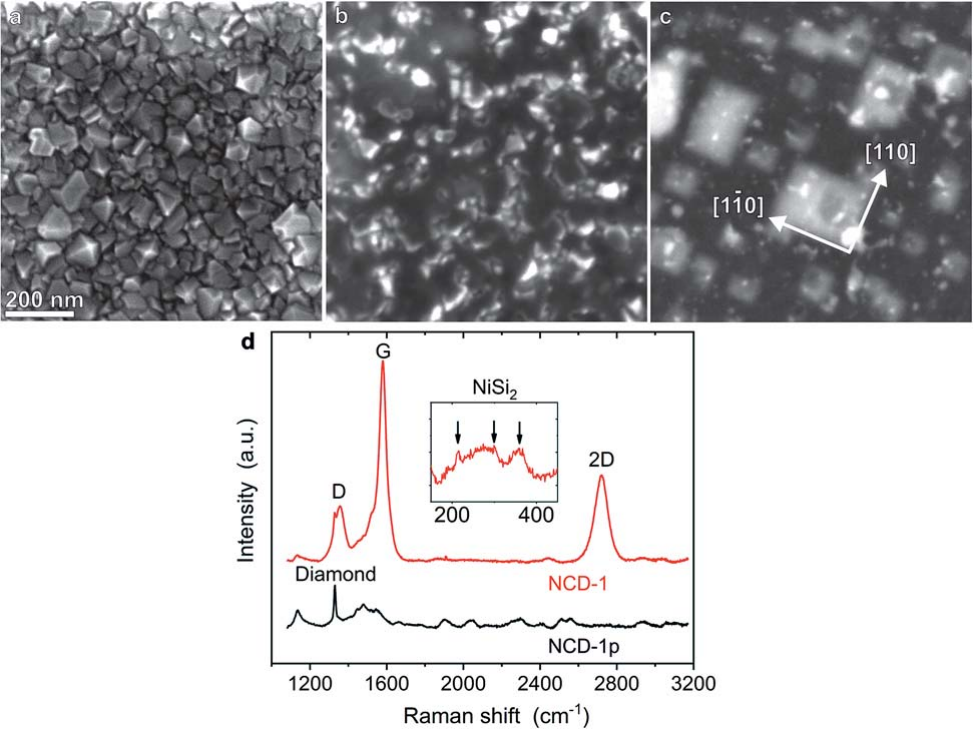
(a) SE SEM image of pristine NCD-1p, (b) SE SEM image and (c) corresponding BSE SEM image of annealed NCD-1. The same scale is used for all three SEM images. (d) Raman spectra of the pristine NCD-1p (black curve) and the processed NCD-1 samples (red curve) acquired with 488 nm excitation wavelength. Inset: Low wavenumber Raman shift region measured with 633 nm excitation wavelength shows weak peaks of NiSi_2_.

Fig. 1a shows a secondary electron (SE) scanning electron microscopy (SEM) image of the pristine NCD sample denoted as NCD-1p. The crystallographic facets of the randomly oriented nanocrystals are well developed. The thickness of the NCD layer determined from an SEM image in a cross-sectional geometry (not shown here) yielded a value of ∼70 nm. After being coated with 20 nm thick Ni layer and heated to 1073 K (10^−6^ mbar) for 30 min, a rather different surface morphology is observed for processed NCD-1: a dark continuous layer with only few sharp-edged crystals along the entire surface is displayed in the SE image (Fig. 1b). In Fig. 1c, the corresponding backscattered electron (BSE) image reflects signal from greater sample depth. Bright objects of rectangular shape dominate the image. The edges of the rectangles are oriented along the [11̄0] and [110] directions of the single crystal Si substrate. The high BSE signal intensity indicates the presence of heavier elements than Si in the substrate. Apparently during heating, Ni penetrates below the diamond layer, interacts with the single crystal Si substrate and forms a compound that is epitaxially oriented with respect to the Si wafer. Energy-dispersive X-ray (EDX) analysis (not shown here) and Raman spectroscopy identified the presence of NiSi_2_.

Fig. 1d shows Raman spectra of the pristine NCD-1p (black) and the annealed NCD-1 (red) using the excitation wavelength of 488 nm. Besides less defined wide features, the spectrum of pristine NCD-1p contains a distinct diamond Raman active mode at 1330 cm^−1^ related to the sp^3^-bonded carbon phase,^11^ the Raman spectrum of the processed NCD-1 (red line) is dominated by the signatures of graphite: the G and 2D peaks located at 1579 cm^−1^ and 2718 cm^−1^, respectively, and the dispersive D peak at 1355 cm^−1^ originating from a disordered sp^2^-phase.^12^ The small value of the ratio 2D/G < 0.5 indicates the presence of a relatively thick layer of graphite; however, a small diamond peak is still resolvable. The presence of graphite can explain the continuous surface layer with low SE signal intensity shown in Fig. 1b. Using the excitation wavelength 633 nm we also identified additional weak and broad Raman peaks located between 200 cm^−1^ and 400 cm^−1^ and assigned to NiSi_2_ (inset of Fig. 1d).^13^ These signals were not detected on pristine NCD and are thus likely related to the bright rectangles located below the sample surface and visible only in the BSE image (Fig. 1c).

**Fig. 2:**
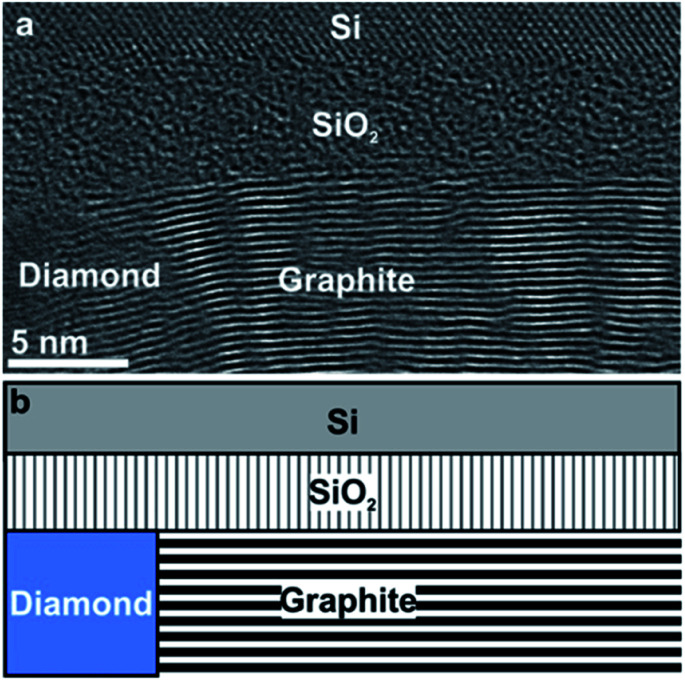
HRTEM image of NCD-1 shows in cross section retained diamond, catalytically converted graphite and the Si substrate with its native SiO_2_ layer. (a) Graphite is grown with the (0001) planes almost perpendicular to the diamond surface and parallel to the Si/SiO_2_ interface. (b) Sketch of the diamond/graphite–SiO_2_/Si configuration seen in (a).

In order to analyse the subsurface structure, TEM investigations of cross-sectional samples have been performed. The TEM image in Fig. 2a shows the arrangement of graphite layers: well-ordered (0001) basal-planes of graphite planes emanate from the diamond grain on the left with an orientation almost perpendicular to the diamond/graphite interface, but parallel to the surface of the substrate. Fig. 2b represents a sketch of the diamond/graphite–SiO_2_/Si configuration imaged in Fig. 2a.

**Fig. 3:**
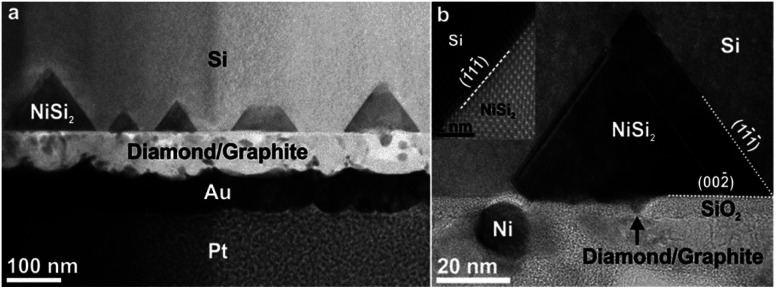
Cross-sectional bright field (BF) TEM images of NCD-1 (BD = [110]). (a) Overview of the Si substrate (top) and its interface with the NCD/graphitic layer. All along the boundary, NiSi_2_ pyramids impinge into the Si substrate. Also shown are the protective Au (dark contrast) and Pt (granular contrast) layers. (b) Detail of a NiSi_2_ pyramid on top of a defective layer of SiO_2_ attached to the NCD/graphite film: On the left a Ni particle is bending the SiO_2_ layer; centred below NiSi_2_, a void in the SiO_2_ layer is marked by an arrow. Inset: A high-angle annular dark-field (HAADF) scanning TEM (STEM) image shows the interface between Si and NiSi_2_ revealing their epitaxial crystallographic orientation relationship.

Such structures appear rather unique. They are attributed to the Ni-mediated catalytic process reported recently.^10^ Upon heating to *T* = 1073 K the thin Ni film undergoes solid-state dewetting and decomposes into nanoparticles. Mobile Ni nanoparticles penetrate along the diamond grain boundaries into the diamond layer and, on their way, catalytically release C atoms from diamond forming graphite. Covalent bonding between diamond and oriented planes of graphite was established during the catalytic transformation due to open dangling bonds, left behind the proceeding Ni particle, which are saturated with the carbon atoms released from the Ni particle. Compared to prior work, there are three distinguishable features in the present case. Although the thickness of deposited Ni was the same (20 nm) in both experiments: (i) the high ratio of graphite relative to the retained diamond; (ii) the well-ordered crystal structure of graphite; (iii) Ni particles are rarely observed in the NCD/graphite layer. These distinguished characteristics are attributed to the lower thicknesses of the NCD (∼70 nm) and SiO_2_ (∼5 nm) layers here, compared to the prior work.^10^ The key influence of these two factors on Ni activity is revealed by the analysis in Fig. 3.

The TEM image in Fig. 3a shows an overview of NCD-1 in cross-section. Protective layers of Au and Pt, as required for focused ion beam (FIB) preparation, appear darker in the bright-field (BF) image, whereas the layer above comprising retained grains of the NCD film and graphite appear brighter; within the carbon layer we also find a few individual Ni particles appearing darker than carbon in the BF image. Between the carbon layer and the Si substrate there is a more or less continuous 5 nm thin SiO_2_ layer with similar grey value as carbon in the BF image. Above the straight Si/SiO_2_ interface, dark pyramids impinge into the single crystal Si. Still, Fig. 3b shows a disruption of the SiO_2_ layer at the centre of the pyramid (marked by the arrow). We suppose that along such disruptions in the thin SiO_2_ layer, Ni can migrate into the Si substrate. A subsequent reaction with Si then forms the NiSi_2_ single crystal pyramids, chemical composition of which has been identified by Raman and EDX spectroscopy. The NiSi_2_ pyramids show almost a perfect epitaxy and a well-defined orientation relationship with the Si substrate. Their bases, appearing as rectangles in the BSE SEM image in Fig. 1c, are oriented parallel to the (001) plane, and the sides parallel to {111} planes of the silicon crystal. The STEM image in the inset of Fig. 3b demonstrates this relationship between the NiSi_2_ nanocrystals and the Si single crystal lattice. The atomic structure of NiSi_2_/Si system has been a subject to TEM and STEM investigations for some time.^14–18^ The surface reactivity of Ni with Si forming epitaxial NiSi and NiSi_2_ within a Si crystal was already explained by a mechanism of a solid-state reaction.^19–23^ More recent publications^24–26^ describe a similar process also in the presence of native SiO_2_ separating Ni films from (001) silicon substrates. They concluded that, since the energy barrier for metal diffusion through non-stoichiometric native SiO_2_ is relatively low,^24,25^ Ni can penetrate through and react with Si forming a NiSi_2_ crystal. The mechanism of decomposition of native SiO_2_ after thermal treatment was closely studied in several works.^25–27^ When temperatures reach 1173 K, Si at the SiO_2_/Si interface starts reacting with the thin SiO_2_ film to form SiO vapor. The SiO molecules leave the surface reducing the density of SiO_2_. Consequently, the generation of voids allows the exposure of the Si surface to Ni. Such a supposed void channel through the SiO_2_ layer is marked below the centre of one of the NiSi_2_ pyramids in Fig. 3b.

### Structural and chemical analysis of NCD-2

**Fig. 4:**
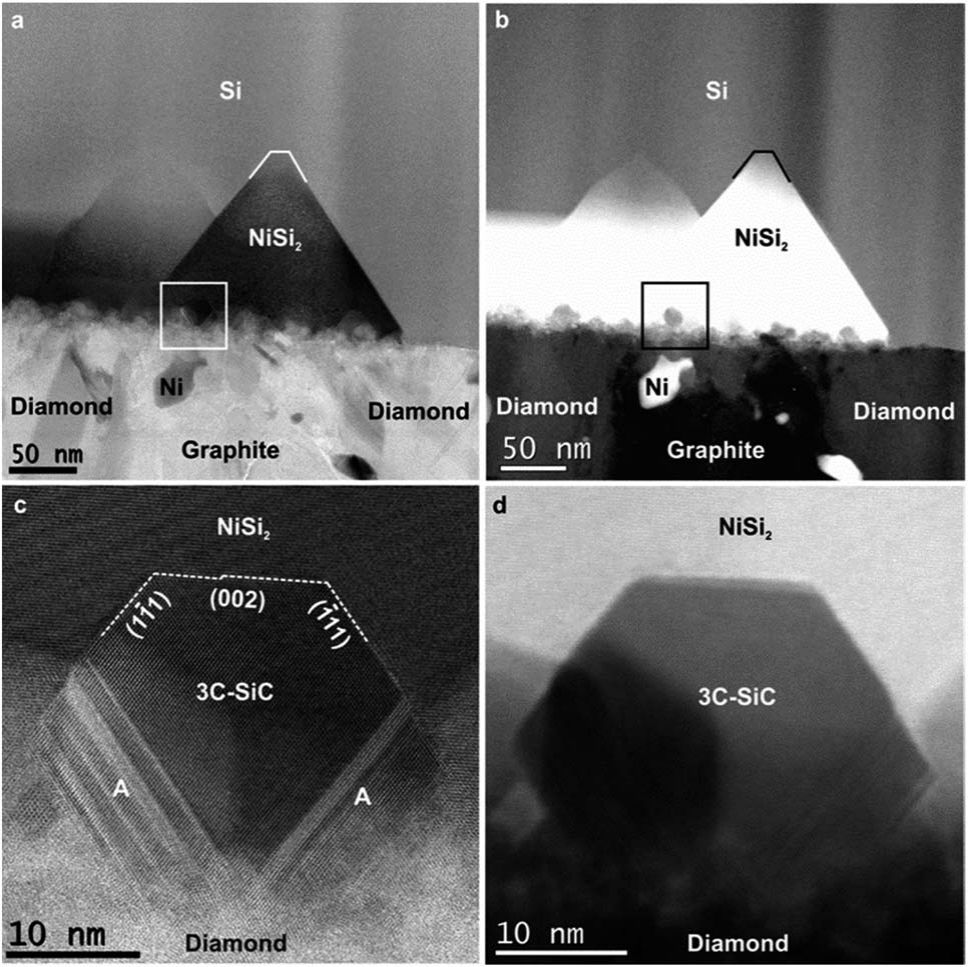
Cross-sectional STEM images of NCD-2. (a) BF and (b) HAADF images show the retained NCD/graphite layer with some embedded Ni nanoparticles below and Si with embedded NiSi_2_ pyramids and additional layer consisting of 10–30 nm sized grains above the interface. (c) BF and (d) corresponding HAADF image of a nanocrystal with a 3C-SiC cubic phase.

As compared to NCD-1, an even more complex set of solid-state reactions is found in NCD-2. The interface between C and Si layers in NCD-2 is shown in STEM images (Fig. 4). Corresponding overview images in BF mode (Fig. 4a) and in HAADF mode (Fig. 4b) display the C layer at the bottom, and the densely overlapping NiSi_2_ pyramids impinging Si above. Unlike in the case of NCD-1, we recognize additional small, yet well-faceted crystals at the bases of NiSi_2_ pyramids.

The HAADF signal of samples increases with the atomic number of their chemical elements and the sample thickness. The small crystals at the pyramid bases appear brighter than carbon below but darker than NiSi_2_ (Fig. 4b and d). EELS confirms the composition of the crystals as SiC; whereas HRTEM images reveal the lattice structure of cubic 3C-SiC; frequently stacking faults and nanotwins are visible in 3C-SiC adjacent to diamond (indicated with A in Fig. 4c). HRTEM also shows a well-defined crystallographic orientation relationship of the SiC nanocrystals with the NiSi_2_ lattice structure (their (002) and {111} planes are almost parallel to each other, Fig. 4c). However, while SiC crystals show well-developed facets in contact with NiSi_2_, their interface geometry with C phases appears rather diffused.

### Composition of the C/Si interfaces

**Fig. 5:**
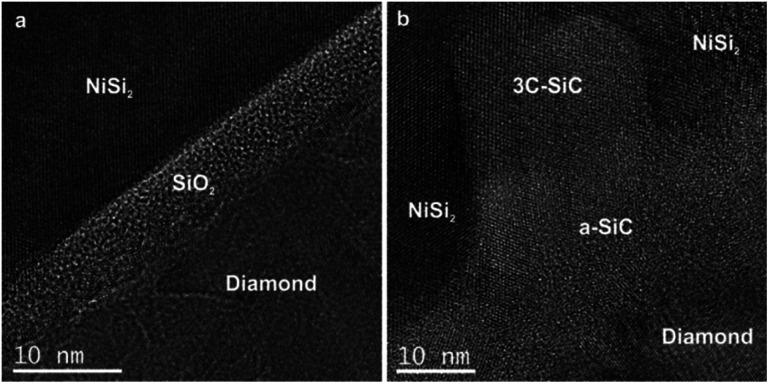
HRTEM images of the phases occurring near the base of NiSi_2_ pyramids formed in samples (a) NCD-1 and (b) NCD-2.

**Fig. 6:**
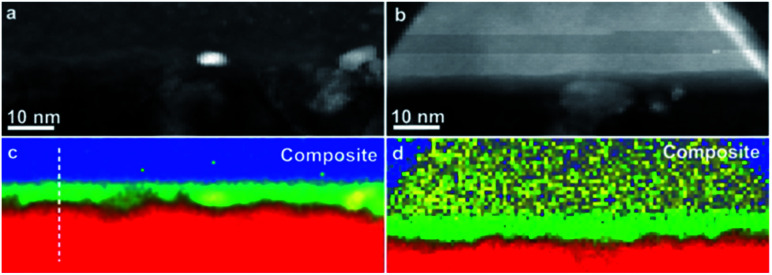
(a and b) HAADF STEM images of two interfacial areas between the Si substrate and the carbon layer in NCD-1; (c and d) superposition of EELS maps of K-edges of Si (blue), C (red), O (green), and Ni L-edge (yellow). The white dashed line in (c) marks the position of the profile shown in Fig. 8a.

**Fig. 7:**
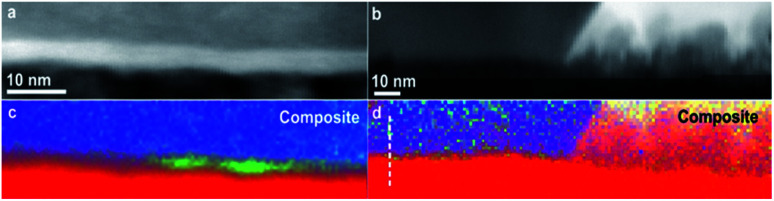
(a and b) HAADF STEM images of two interfacial areas between the Si substrate and the carbon layer in NCD-2; (c and d) superposition of EELS maps of K-edges of Si (blue), C (red), O (green), and Ni L-edge (yellow). The white dashed line in (d) marks the position of the profile shown in Fig. 8b.

The HRTEM image of the NCD-1 sample in Fig. 5a clearly shows a 4–7 nm thick layer with a granular contrast identified as amorphous SiO_2_, which separates a faceted NiSi_2_ pyramid (top) and diamond (bottom). Contrastingly, sample NCD-2 displays a rather complex geometry of NiSi_2_ (top) in contact with the carbon layer (bottom), which are separated by an irregularly bounded cubic SiC crystal that impinges the NiSi_2_ pyramid (Fig. 5b). A detailed study of the chemical composition at the C/Si interface in both NCD-1 and NCD-2 samples was performed by EELS mapping. The maps of spectral edges: Si K (blue), C K (red), O K (green) and Ni L (yellow) are imaged in Fig. 6 (for NCD-1) and Fig. 7 (for NCD-2). A similar analysis was carried out at the C/NiSi_2_ interfaces.

The composite images in Fig. 6c and d show a superposition of EELS maps of K-edges of Si (blue), C (red), and O (green) as well as the Ni L-edge (yellow). They correspond to two different interfacial areas shown as HAADF STEM images in Fig. 6a and b, respectively. The left panels (Fig. 6a and c) display the diamond/Si interface, whereas the right panels (Fig. 6b and d) the diamond/NiSi_2_ interface. Besides some Ni nanoparticles that have penetrated into the interfacial layer, both areas show a rather continuous green band in the EELS map related to the O K-edge, indicating that O is present all along the Si/C interface of NCD-1.

The situation is different in the sample NCD-2 shown in Fig. 7. There, the EELS map of the Si/C interface indicates the limited presence of O, which is visible only in two localized green spots in Fig. 7c. This result opposes the TEM image in Fig. 7a which displays a layer of continuous bright contrast between Si and C. This case is even more pronounced in the location near and below a NiSi_2_ pyramid in Fig. 7b and d where the O K signal is missing at all.

**Fig. 8:**
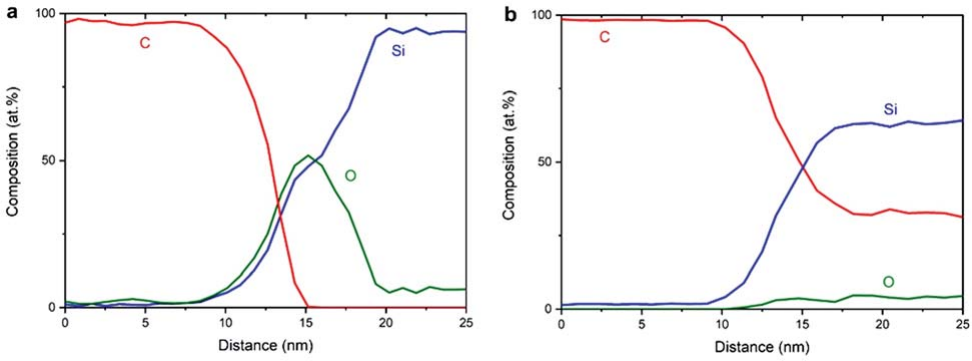
EELS spectral intensities of C, Si and O (a) for NCD-1 along the dashed line in Fig. 6c and (b) for NCD-2 along the dashed line in Fig. 7d.

Fig. 8a shows the EELS spectral intensity profiles using data of individual maps of C, Si and O from sample NCD-1 plotted *versus* distance along the dashed line marked in Fig. 6c. The C signal (red line) sharply vanishes at the distance where the O signal (green line) reaches the maximum following the shoulder of the increasing Si signal. This quantitative figure suggests that the stoichiometry in the centre of the interfacial oxide layer rather corresponds to SiO (50% Si : 50% O) than to SiO_2_. In any case, it is concluded that an oxide layer is preserved in sample NCD-1.

In the case of NCD-2 the EELS spectral intensity profiles (Fig. 8b) of C, Si and O plotted *versus* distance along the dashed line in Fig. 7d shows that O is almost absent in the centre of the interfacial layer, while a small O signal (∼5%) was detected in the entire area constituted by Si indicating some surface oxidation of the FIB lamella. At the Si/C interface, the C signal (red line) drops to about 30% and then stays nearly at a constant value. The C signal almost complements the Si signal which indicates the interdiffusion of C and Si. More importantly, in the centre of the interface, the Si and C intensities are approximately equal, indicating the presence of a SiC phase. Together with the HRTEM image contrast, which points to the presence of an amorphous layer between C and Si (see Fig. 5b), it is concluded that amorphous a-SiC formed.

Based on the data shown in Fig. 5a, 6c and 8a, it is concluded that crystalline SiC cannot have formed in sample NCD-1 since a continuous oxide layer attached to Si is still preserved after the MWPECVD and following heat treatment (see Tables 2 and 3). Despite being defective (Fig. 3b and 6c) and rather thin (∼5 nm, Fig. 5a, 6c, and 6d), the SiO_2_ layer with reduced amount of O to 50% (corresponding to SiO) still seems to be a sufficiently strong barrier for the diffusion of C (see Fig. 8a). Nevertheless, such an interfacial barrier does not prevent Ni to penetrate through and react with the Si to form NiSi_2_ (Fig. 3 and 6). The question arises, why crystalline SiC occurs in NCD-2 at the base of the NiSi_2_. We suppose that two major differences between the samples NCD-1 and NCD-2 play an important role in the formation of crystalline SiC. Firstly, there are significant differences between the process conditions during the MWPECVD growth of NCD-1p *versus* NCD-2p (see Table 2), namely the higher plasma power, pressure and temperature and longer time during the NCD-2p thin film synthesis which may have more destructive effect on the initial SiO_2_ layer, forming an intermixed C/Si layer (a-SiC) instead (Fig. 8b).^28–30^ Since no SiC crystals have formed outside NiSi_2_ pyramids (Fig. 4 and 7), NiSi_2_ seems to catalytically trigger the reaction of C with Si, forming crystalline SiC. At the annealing temperature, this might occur by the crystallization of the amorphous a-SiC layer (for Si-rich a-SiC, crystallization was reported to already occur at a temperature of 1173 K (ref. 31)). Crystalline nuclei of SiC might form at the a-SiC/Si interface and subsequently grow into NiSi_2_ by a Ni-triggered catalytic reaction of the present Si and C which diffuse into the pyramids.

**Fig. 9:**
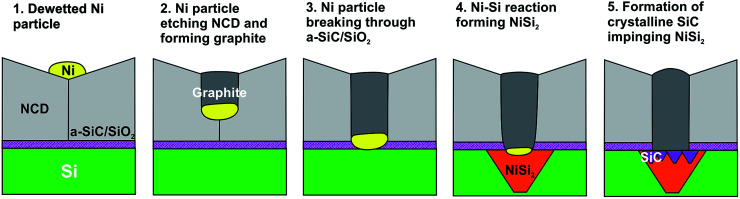
Scheme of the Ni–C, Ni–Si and Si–C solid-state reactions leading to NiSi_2_ and SiC formation at the initial Si/NCD interface in NCD-2.

The observed chain of solid-state reactions between Ni–C, Ni–Si, and Si–C in sample NCD-2 is sketched in Fig. 9, where Ni contributes to transform diamond to graphite, reacts with Si to form NiSi_2_ and activates crystallization of a-SiC. Reduced to the steps 1 to 4, a similar model can be also applied for the reactions in the NCD-1 sample in cases where the SiO_2_ layer is defective.

### Electrical transport

**Fig. 10:**
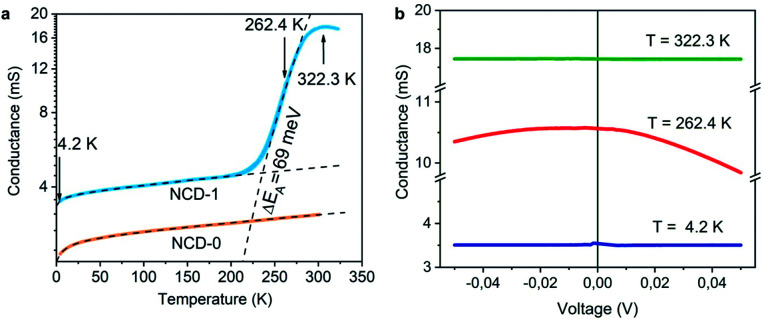
(a) The temperature dependence of conductance *G*(*T*) of NCD-1 (blue curve) in comparison with *G*(*T*) of “NCD-0” published in ref. 10 (orange curve) plotted in semi-logarithmic scale. Both measured curves are fitted by eqn (1) (black dashed curves), whereas the steep region in the *G*(*T*) of NCD-1 is fitted by eqn (2). (b) Voltage dependence of conductance *G*(*V*) of sample NCD-1 measured at *T* = 4.2 K (dark blue), 262.4 K (red), and 322.3 K (green) exhibiting different conductance regimes.

The electrically insulating properties of diamond and conductive properties of graphite offer a possibility to evaluate the relative amount and percolation of graphite derived from transformation of NCD. We measure, therefore, the dependence of electrical conductance on the temperature (*G*(*T*)) and on the electrical field (*G*(*V*)). Plotted in a semi-logarithmic scale, Fig. 10a presents the results of *G*(*T*) for NCD-1 (blue curve) acquired during cooling from room temperature down to 4.2 K followed by heating to 322 K in the 4-probe configuration; the two curves overlap without hysteresis indicating a correct measurement, though the overall *G*(*T*) behaviour is unusual.

**Fig. 11:**
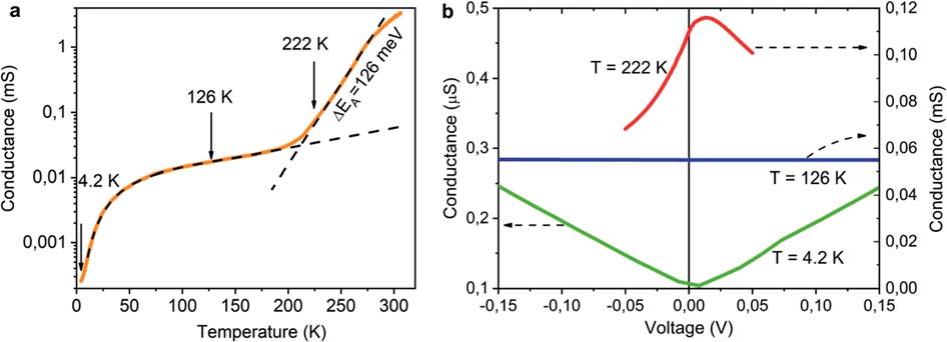
(a) *G*(*T*) of NCD-2 plotted in a semi-logarithmic scale. (b) Voltage dependence of conductance measured at *T* = 4.2 K (green), 126 K (blue), and 222 K (red) showing different conductance regimes.

At low temperatures, the conductance of NCD-1 shows only a very moderate increase with rising temperature but, at about 220 K, the *G*(*T*) slope steepens and culminates in a maximum of *G*(*T*) at *T* ∼ 300 K. For comparison, the *G*(*T*) curve published in ref. 10 (orange curve) is added to Fig. 8a, which was measured for a NCD/graphite layer (here denoted as NCD-0) that is separated from the Si substrate by a 1500 nm thick SiO_2_ layer; such a thick oxide layer prohibited other reactions than the Ni-catalysed graphitization of diamond. At temperatures *T* < 200 K, the blue and orange experimental curves follow the same trend in conductance; however, while the orange curve of NCD-0 continues with the same trend for the whole temperature range, the blue curve of NCD-1 above 200 K strongly deviates from the low-temperature curve. This fact serves as evidence that the steep conductance slope seen in NCD-1 is unrelated to the electronic transport in the graphite layer itself. The voltage dependences of conductance *G*(*V*) (Fig. 10b) were measured at three constant temperatures 4.2, 262.4, and 322.3 K, which were chosen from the three distinct conductance regimes of *G*(*T*). While *G*(*V*) is constant at *T* = 4.2 and 322.3 K, *G*(*V*) exhibiting the ohmic behaviour, at the intermediate temperature of 262.4 K (steepest slope section of *G*(*T*)), *G*(*V*) is not constant but declines with increasing voltage of both polarities. The TEM images of the NCD-1 cross-section in Fig. 4 offers an explanation for the temperature-activated breakdown of the electrical current from the graphite percolating network through the defective SiO_2_ layer down to boron-doped Si substrate and NiSi_2_, which both are electrically well-conductive. Since the electrode system is fabricated on top of the NCD/graphite layer, the current passes the interface twice: from graphite to Si substrate and back to graphite. The measured *G*(*T*) curve for the NCD-2 sample is plotted in the semi-logarithmic scale as well (Fig. 11a). It shows a similar temperature-dependent electrical breakdown despite of the even more complex interfacial structure, where a part of the native SiO_2_ has been transformed into electrically insulating SiC.

Despite the conductance value of NCD-2 being a few orders of magnitude lower than that of NCD-1, the sudden *G*(*T*) increase similarly starts above 200 K (Fig. 10a). *G*(*V*) acquired from NCD-2 (Fig. 11b) demonstrates three different conduction mechanism regimes along the measured temperature range. At *T* = 4.2 K, *G*(*V*) behaves like a semiconductor with an energy gap, where current is activated by the electric field; the conductance is, therefore, enhanced by the increasing bias voltage. At *T* = 126 K, we identify the ohmic regime with a constant value of conductance. Finally, at *T* = 222 K, *G*(*V*) shows a bell-like shape typically understood in terms of hot carriers dissipating their energy in form of acoustic phonons. As the carriers gain kinetic energy sufficient to overcome the potential barrier across the dielectric interface, charge transport is dominated by the leakage current through the Si substrate. Nevertheless, while overcoming the interfacial barriers, the charge carriers transfer a part of their kinetic energy to lattice vibrations; the energy dissipation is seen as a decrease of *G*(*V*) with increasing electric field above a temperature of 200 K. The experimental results are fitted by models of conduction mechanisms considered for different temperature regions. At low temperature, the conductance through the network of percolating graphitic domains follows an expression for fluctuation-assisted tunnelling (FAT)^32–34^ mechanism of electronic transport:1
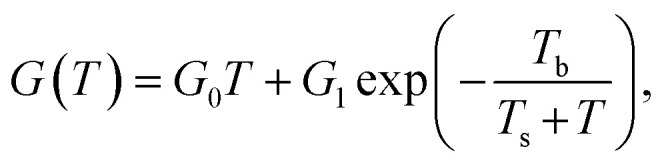
where *G*_0_ is a constant (in units S K^−1^) and *G*_1_ is a prefactor of the residual conductance at *T* = 0 K, *T*_b_ characterizes the tunnelling energy barrier between conductive regions, and *T*_s_ determines the tunnelling efficiency at *T* = 0 K. In the temperature region above 200 K, where electrical breakdown takes place, the Arrhenius formula considering the activation energy to overcome the interfacial barrier is used to fit the steep increase of *G*(*T*):^35^2
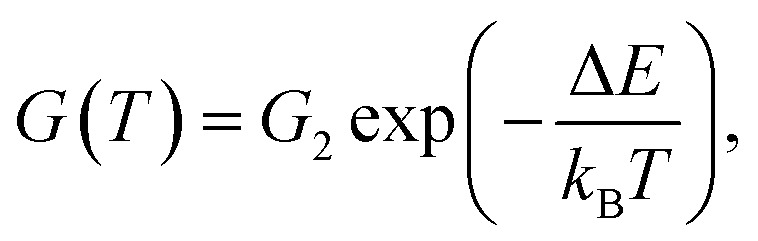
where *G*_2_ is a constant, Δ*E* is the activation energy and *k*_B_ is the Boltzmann constant. The particular temperature of the breakdown event (200 K) can be well correlated with an observed anomaly in *G*(*T*) of NiSi_2_ reported in literature^36,37^ and attributed to stress in the NiSi_2_ structure.

The values of the fitting parameters for NCD-1, NCD-2 as well as for already published NCD-0 are summarized in Table 1.

**Table 1:** Fitting parameters to eqn (1) and (2)

Sample	*G*_0_ (S)	*G*_1_ (S)	*k*_B_ *T*_b_ (meV)	*T*_s_ (K)	Δ*E* (meV)
NCD-1	3.60 × 10^−6^	3.72 × 10^−3^	0.059	6.42	69
NCD-2	7.01 × 10^−6^	3.10 × 10^−5^	7.599	11.85	126
NCD-0 (ref. 10)	2.14 × 10^−6^	2.46 × 10^−3^	0.170	9.48	—

The fitting parameters in Table 1 extracted from the electronic transport model corroborate our results from electron microscopy. The structure and compositional characteristics of two NCD samples differ due to various solid-state reactions taking place when the experimental setup is varied. The FAT term well reflects the crystal order of the graphite domains with a very small tunnelling barrier (*k*_B_*T*_b_ = 0.059 meV) for NCD-1 showing a highly crystalline graphite structure (Fig. 2), whereas a 12-times larger tunnelling barrier (*k*_B_*T*_b_ = 7.599 meV) is found for NCD-2 with disordered graphite domains and much smaller relative portion of graphite in the diamond layer. The value of the activation energy Δ*E* extracted from the fitting to eqn (2) for the thermally activated electrical breakdown yielded 69 meV for NCD-1, whereas a two times higher activation energy (126 meV) is obtained for NCD-2 with insulating SiC between the Si substrate and the carbon layer.

## Experimental

### Sample preparation

Two NCD films with average thicknesses of ∼70 nm and 250 nm, denoted as NCD-1p (pristine) and NCD-2p, respectively, were grown by focused microwave plasma enhanced chemical vapor deposition (MWPECVD) on single-crystal Si(001) substrates covered with a ∼5 nm native SiO_2_ layer. The most important parameters (film thickness, MWPECVD process time, pressure, microwave power, and temperature) of the NCD growth are summarized in Table 2.

**Table 2:** MWPECVD process parameters for the pristine NCD films on Si substrates with a 5 nm native oxide layer

Sample	NCD thick. [nm]	Time [min]	Pressure [mbar]	Power [W]	Temperature [K]
NCD-1p	70	24	60	3000	1050
NCD-2p	250	60	100	4500	1250

In the next step, both samples NCD-1p and NCD-2p were covered with a 20 nm thick Ni film by using thermal evaporation. These samples were further subjected to a heat treatment to initialize the catalytic graphitization of the diamond surface and denoted as NCD-1 and NCD-2. The NCD-1 sample was annealed at 1073 K in vacuum (10^−7^ mbar) for ∼30 min (details in ref. 9), whereas the NCD-2 sample was annealed at 1173 K in a forming gas atmosphere (95% Ar/5% H_2_) in Carbolite tube furnace. Table 3 summarizes process parameters of the heat treatments.

**Table 3:** Process parameters of annealing NCD films covered with a 20 nm thick Ni layer

Sample	Temperature [K]	Pressure [mbar]	Heat. time [min]
NCD-1	1073	10^−7^	30
NCD-2	1173	13 (Ar + 5% H_2_)	10

### Characterization

Raman spectroscopy was carried out using a WITec alpha 300 RA confocal microscope with 488 nm and 633 nm excitation wavelengths. A 100×/0.9 objective lens and 1200 g mm^−1^ grating were used for the measurements. Spectra were collected at room temperature for about 1 min, keeping the laser power at 4 mW.

For SEM, a Zeiss Supra 55 VP instrument equipped with a SE InLens and BSE detectors was used at 10 kV accelerating voltage.

Cross-sectional samples for (S)TEM were prepared by FIB using a FEI Quanta 3D FEG instrument. Using electron backscatter diffraction methods, the crystallographic orientation of the FIB lamella was carefully selected to run parallel to a (110) plane normal to the (001) surface of the Si substrate. Protective layers of Au (100 nm) and Pt (2 μm) were deposited onto the sample surface before FIB preparation to protect the sample surfaces from beam damage. Firstly, foils were thinned down to ∼40 nm with Ga ions at 30 kV accelerating voltage, and finally cleaned at 5 and 2 kV.

HRTEM and STEM was done by using JEOL JEM ARM200F (S)TEM operated at 200 kV equipped with Cs correctors for both objective lens and the condenser system. In the case of TEM, bright-field (BF) images were obtained. In the case of STEM, images were taken using a BF and HAADF detector. A double-tilt holder was used to orientate the beam direction (BD) along the [110] zone axis of the Si substrate bringing its (001) surface in an edge-on orientation. EELS images were acquired with a Gatan imaging filter (GIF) 965 Quantum ER DualEELS (convergence and collection semi-angle were 29 mrad and 74 mrad, respectively). The energy spread of the electron beam of 0.45 eV was determined from the full-width at half maximum of the zero-loss peak; the detector energy dispersion was set to 1.0 eV per channel. A dwell time per pixel of 50 ms was set and summed *via* 400 cycles for spectrum acquisition. Spectral images were acquired with a size of 127 × 27 pixels at a pixel size of 0.53 nm with an exposure time of 20 ms per pixel and 15 cycles.

Electrical transport measurements were performed in the four-probe configuration. Au electrodes were evaporated onto the samples and wire-bonded to Kyocera chip-carriers. The chip-carrier was located at the bottom end of a long rod with Si-diode temperature sensor (DT-470) and a resistive heating coil. The rod was placed into a vacuum-tight tubular chamber filled with He gas; an attached elastic balloon secured the atmospheric pressure of He in the chamber. A source-meter Keithley 2635B was supplying constant currents of 1–100 μA and collecting the voltage data. The sample temperature was controlled by immersing the tubular chamber with the sample at the bottom into the liquid He (LHe) container, utilizing the temperature gradient above the LHe level from 4.2 K to 300 K. The temperature of the sample was calibrated to the resistance of the Si-diode DT-470 supplied by constant current 1 μA while the voltage drop was measured by multimeter Keithley 2000. *I*–*V* curves at selected temperatures were acquired by sweeping bias voltage between ±0.05 V for NCD-1 and ±0.15 V for NCD-2.

## Conclusions

We studied Ni-mediated phase transformations of thin nanocrystalline diamond films deposited by MWPECVD on Si wafers covered with a layer of native SiO_2_. We identified specific processes that arise when different conditions are used for MWPECVD synthesis: at relatively moderate plasma conditions, a continuous oxide layer between NCD and the Si substrate is preserved, whereas, at high-power plasma conditions, SiO_2_ transformed to a layer of amorphous a-SiC. After thermal annealing of Ni-coated samples, microscopic and spectroscopic investigations complemented by measurements of the electrical conductivity revealed the presence of new phases that were produced by Ni-mediated reactions: graphite covalently attached to diamond grains, NiSi_2_ crystals impinging the Si substrate and SiC crystals impinging NiSi_2_. The key factor for these reactions was the permeability of the SiO_2_ and the a-SiC interface layers to Ni. While NiSi_2_ was formed in both cases, the formation of the 3C-SiC phase was observed only when the SiO_2_ layer was absent and NiSi_2_ was present. We propose a model that describes step-by-step the reaction processes during the thermal treatment of the Ni/NCD/Si system. Results of conductivity measurements were explained by two main mechanisms: (i) electric transport through the percolating network of graphite and (ii) electrical current breakdown occurred at *T* ≈ 200 K due to charge carriers leaking from graphite to the NiSi_2_/Si substrate. Different leakage activation energies were calculated for SiO_2_ (Δ*E* = 69 meV) and SiC (Δ*E* = 126 meV) insulating layers at the carbon/silicon interface. The presence of the NiSi_2_/Si epitaxial metal–semiconductor interface combined with graphite that is covalently-attached to diamond across the weakly permeable dielectric interface might be of particular interest for electronic devices.

## Conflicts of interest

There are no conflicts to declare.

## Supplementary Material
